# Analysis of Gene, Environment, and Sex Interaction in the Development of Autistic-like Phenotype in Mice

**DOI:** 10.3390/ijms27062566

**Published:** 2026-03-11

**Authors:** Danielle Santana-Coelho, Grace Porter, Juan Morales, Jason C. O’Connor

**Affiliations:** 1 Department of Pharmacology, University of Texas Health San Antonio, San Antonio, TX 78229, USA; coelhod@uthscsa.edu (D.S.-C.);; 2 South Texas Veterans Health System, Audie L. Murphy VA Hospital, San Antonio, TX 78229, USA

**Keywords:** maternal immune activation, cntnap2, autism, behavior, development

## Abstract

Autism Spectrum Disorder (ASD) is a developmental disorder that manifests a broad variability of phenotypes. The underlying factors contributing to the diverse presentation of autistic phenotypes remain poorly understood. Studies have shown that environmental and genetic factors could contribute to ASD. Additionally, there is a sex bias in the disorder, where the prevalence in males is higher than in females. But it is still unknown how exposure to similar risk factors can lead to different phenotypes. The three-hit theory states that the vulnerability of an individual to develop ASD is modulated by the interplay between genetic predisposition, sex, and environmental insults. To better understand this phenomenon, we investigated whether an environmental insult, via maternal immune activation (MIA) during pregnancy could influence the development of the autistic-like phenotype in a genetically predisposed mouse strain, *contactin-associated protein-like 2* (*CNTNAP2*) knockout. *CNTNAP2* knockout, sex, and maternal immune activation had significantly additive effects on repetitive/stereotyped and social behavior in the offspring, while working memory and sensory gating were not affected by hits. These results indicate that genetics, sex, and environment interact to influence autistic-like phenotypes in a behavior-specific manner.

## 1. Introduction

Autism Spectrum Disorder is a neurodevelopmental disorder with an early onset that affects 1 in every 31 children in the USA. According to the Diagnostic and Statistical Manual of Mental Disorders (DSM-5-TR; 2022), the main characteristics presented by individuals with ASD can include a persistent deficit in social communication and social interactions, repetitive patterns of behavior, and restricted interests. The ASD classification is an umbrella composed of autistic disorder, Asperger’s disorder, and childhood disintegrative disorder, which demonstrates the variability of phenotypes in ASD.

Research into the etiology of ASD has shown that the genetic background can be a significant factor in the development of the disorder, as several mutations have been linked to autism [1,2,3]. Although genetic factors strongly contribute to ASD risk, genetics alone cannot fully explain ASD pathophysiology and phenotypic heterogeneity. For example, despite the high rates of ASD in monozygotic twins, not all identical twins are autistic even when the parents are autistic themselves [4,5], which raises the question of what other factors are responsible for the development of ASD. Studies have identified that several environmental factors, such as prenatal exposure to drugs, infection, and inflammation, are associated with ASD [6,7,8]. Among these, there is growing evidence that links maternal inflammation during pregnancy to an increased risk of ASD development in the offspring [8,9].

Autism presents a spectrum of phenotypes that range from mild to severe symptoms, corroborating the idea that genes and the environment could act synergistically to result in a diverse range of phenotypes. Other than the genetic and environmental etiology of autism, ASD exhibits strong sex bias, with males being four times more affected than females. Additionally, studies show a sex-dependent effect of both genetic [10] and environmental factors [11,12]. Altogether, these observations support a multifactorial model in which genes, environment, and biological sex interact to influence ASD risk, severity, and phenotypic presentation. However, few studies have evaluated how these factors interact within the same experimental framework to affect phenotypical outcomes.

To address this gap, we investigated the three-hit hypothesis, where genotype (*CNTNAP2* knockout), sex, and environmental insult via maternal immune activation can interact to develop a more severe autistic-like phenotype. We focused on the *CNTNAP2* gene that encodes a protein that is involved in the clustering of K+ channels in myelinated axons [13]. The *CNTNAP2* knockout mice have been used as a model of mild autistic-like phenotype in rodents [14,15], and knockout mice for this gene have been shown to present with an altered inflammatory state [16]. The environmental insult chosen was the model of MIA through the administration of Polynosinic:polycytidylic acid (Poly IC) that has been extensively used to investigate the role of maternal inflammation in the disruption of neurodevelopment in the offspring [7,17]. Here, we hypothesized that maternal immune activation may have a cumulative effect with the lack of *CNTNAP2* and sex, generating a more severe phenotype when the susceptibility factors are compounded versus when the offspring is exposed to only one factor in isolation. To test this hypothesis, we performed a battery of behavior tests, which included the assessment of repetitive and stereotyped behavior, deficits in communication, working memory, sociability, and sensory gating.

## 2. Results

### 2.1. Immune Response 24 h After Poly IC in the Maternal Plasma

The levels of cytokines and chemokines were measured in maternal plasma 24 h after Poly IC to confirm an immune response in both genotypes. The levels of IFN-γ, IL-1β, IL-4, IL-6, IL-10, MCP-1, and TNF-α were not significantly high at the 24 h timepoint (*p* > 0.05, Table 1). IP-10 was the only chemokine that was still significantly elevated at 24 h (F_1,10_ = 6.68, *p* < 0.05). There were no differences in the immune responses between the genotypes (F_1,10_ = 0.13, *p* > 0.05, Table 1).

### 2.2. Three Hits Decreased the Number of Vocalizations Performed by the Pups

**Figure 1:**
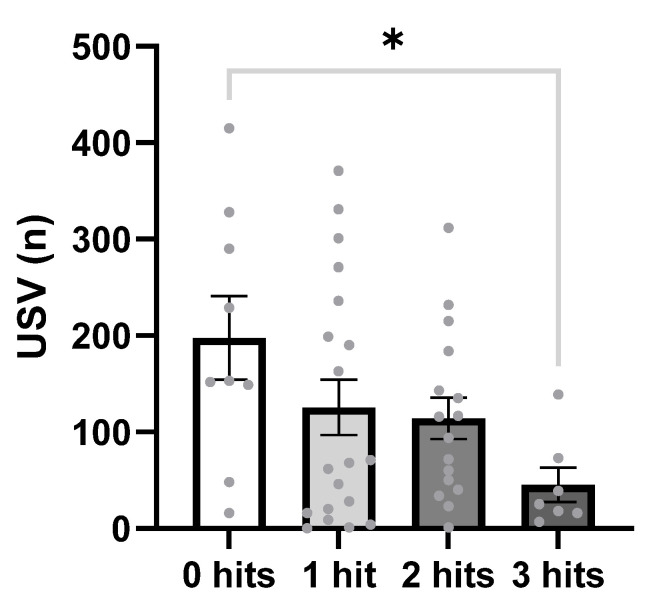
The number of isolation-induced ultrasonic vocalizations decreased in response to 3 hits. The total number of ultrasonic vocalizations performed by the pups in the isolation-induced vocalization test was quantified at postnatal day 7. 0 hits *n* = 9, 1 hit, *n* = 19, 2 hits *n* = 16, 3 hits *n* = 7. Data is represented as mean ± SEM and was analyzed using a one-way ANOVA test with Tukey’s post hoc test. * *p* < 0.05.

Deficits in communication were evaluated in the isolation-induced ultrasonic vocalization test. One-way ANOVA revealed a significant decrease in the number of vocalizations in response to hits (F_3,43_ = 2.85, *p* = 0.048). Post hoc analysis showed that three hits significantly decreased the number of USVs when compared to the 0-hit group (*p* = 0.029, Figure 1). To better understand how each factor affected the output of the behavior paradigms, we also analyzed the raw data with a three-way ANOVA with genotype, sex, and treatment as factors. An analysis of raw data identified that Poly IC significantly decreased the number of vocalizations in all groups (F_1,43_ = 6.57, *p* = 0.0206, Supplementary Table S1).

### 2.3. Stereotyped and Repetitive Behavior Is Affected by Hits in a Behavior-Test-Dependent Manner

**Figure 2:**
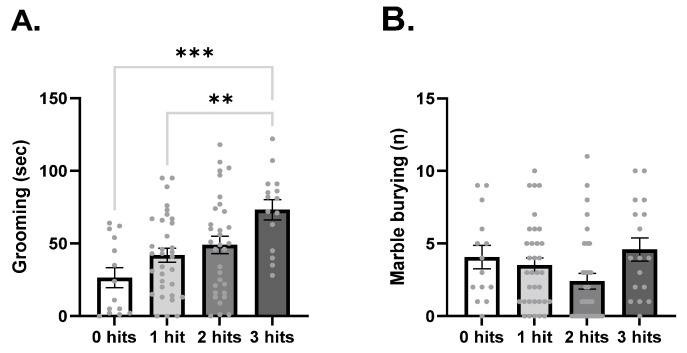
Stereotyped and repetitive behavior was altered in a hit- and behavior-dependent manner. (**A**) Time spent grooming. 0 hits *n* = 14, 1 hit, *n* = 33, 2 hits *n* = 32, 3 hits *n* = 15. (**B**) Total number of marbles buried. 0 hits *n* = 14, 1 hit *n* = 34, 2 hits *n* = 32, 3 hits *n* = 16. Data was analyzed using a one-way ANOVA test with Tukey’s post hoc test. ** *p* < 0.01; *** *p* < 0.001.

One of the main features of ASD is stereotyped and repetitive behavior. To evaluate if the number of hits could influence the severity of this phenotype, we performed the grooming and marble-burying tests. Time spent grooming was increased by the number of hits (F_3,90_ = 6.57, *p* = 0.0005; Figure 2A). The animals affected by three hits spent a significantly longer time grooming when compared to 0 hits (*p* = 0.0003) and one hit (*p* = 0.0056). Marble-burying behavior was not significantly affected by hits (F_3,93_ = 2, *p* = 0.0905, Figure 2B).

Raw data analysis identified a main effect of genotype (F_1,85_ = 44.81, *p* < 0.0001, Supplementary Table S1) characterized by increased time spent grooming in *CNTNAP2*−/− mice. Three-way ANOVA was performed and identified a main effect of genotype (F_1,89_ = 12.20, *p* = 0.007), treatment (F_1,89_ = 6.10, *p* = 0.0154), and an interaction between genotype, sex, and treatment (F_1,89_ = 3.97, *p* = 0.0493; Supplementary Table S1). Post hoc analysis showed no significant difference (*p* > 0.05).

### 2.4. Hits Did Not Alter Performance in the Y-Maze Test

**Figure 3:**
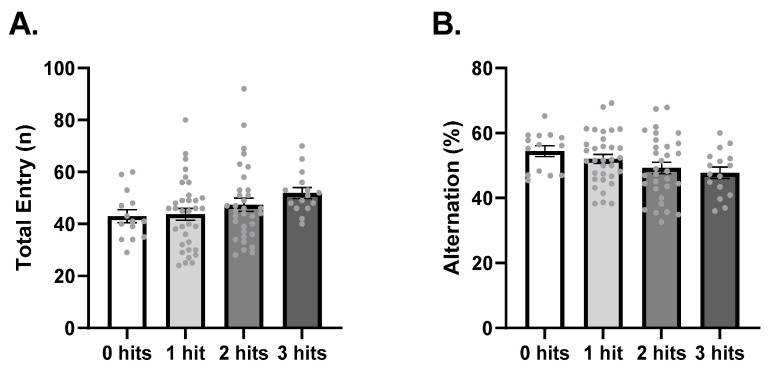
Spontaneous alternation in the Y-maze is not dependent on the number of hits. (**A**) Total number of arm entries. 0 hits *n* = 14, 1 hit, *n* = 34, 2 hits *n* = 33, 3 hits *n* = 15. (**B**) Percentage of spontaneous alternation. 0 hits *n* = 14, 1 hit, *n* = 33, 2 hits *n* = 31, 3 hits *n* = 17. Data is expressed as mean ± SEM and analyzed using the one-way ANOVA test.

Eight-week-old mice were tested in the Y-maze test as a measure of working memory and insistence on sameness. An analysis of the total number of entries in the Y-maze arms identified no effect of hits (F_3,92_ = 1.83, *p* = 0.1479, Figure 3A). The percentage of alternation presented by the animals was also not affected by hits (F_3,90_ = 2.21, *p* = 0.0928; Figure 3B).

Raw data analysis showed that the total number of entries was affected by an interaction between genotype and sex (F_1,85_ = 4.36, *p* = 0.0397). the post hoc test showed that *CNTNAP2*−/− had a higher number of entries when compared to WT Saline male mice, independent of the treatment (*p* > 0.05). Furthermore, a main effect of genotype was identified, showing that *CNTNAP2*−/− had a higher number of entries than wild-type mice (F_1,85_ = 28.42, *p* < 0.0001; Supplementary Table S1). Additionally, male *CNTNAP2*−/− Poly IC mice presented more entries than male WT Poly IC (*p* = 0.0102). A main effect of genotype identified that *CNTNAP2*−/− mice performed fewer alternations than wild-type mice (F_1,86_ = 11.50, *p* = 0.0011; Supplementary Table S1).

### 2.5. Offspring’s Social Preference Increased with Hits

**Figure 4:**
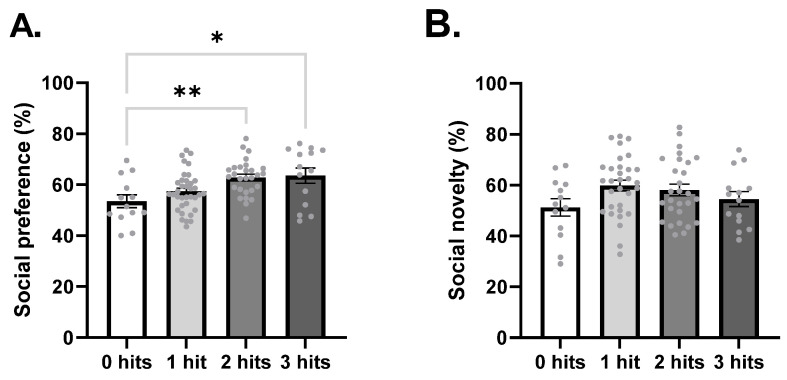
Social preference was affected by hits. (**A**) Percentage of social preference. 0 hits *n* = 13, 1 hit, *n* = 36, 2 hits *n* = 28, 3 hits *n* = 14. (**B**) Percentage of social novelty preference. 0 hits *n* = 13, 1 hit, *n* = 31, 2 hits *n* = 28, 3 hits *n* = 14. Data is expressed as mean ± SEM and analyzed using the one-way ANOVA test with Tukey’s post hoc test. * *p* < 0.05 and ** *p* < 0.01.

The three-chamber test was performed at 8 weeks of age to detect deficits in sociability. In the first part of the test, social preference was calculated and analysis of variance (ANOVA) showed that social preference increased with hits (F_3,87_ = 5.63, *p* = 0.0014, Figure 4A). Post hoc analysis identified a significant difference between two hits (*p* = 0.0069) and three hits (*p* = 0.0119) when compared to 0 hits. Social novelty preference, however, was not affected by hits (F_3,82_ = 1.88, *p* = 0.1391, Figure 4B).

A main effect of sex and treatment was identified for social preference. Poly IC increased social preference (F_1,83_ = 5.60, *p* = 0.0203). Females presented a lower social preference index than males (F_1,83_ = 4.55, *p* = 0.0358, Supplementary Table S1). An interaction between treatment and sex was identified for social novelty (F_1,78_ = 4.95, *p* = 0.0053). Post hoc analysis identified a statistically significant difference between males and females for wild-type control (*p* = 0.0171; Supplementary Table S1).

### 2.6. Hits Did Not Affect Baseline Startle Response or Prepulse Inhibition

**Figure 5:**
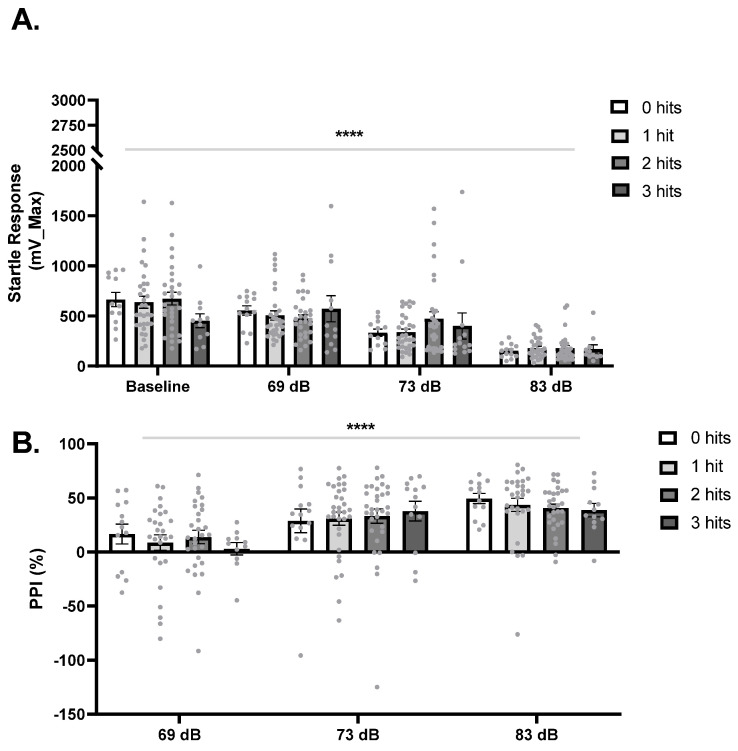
Hits had no effect on prepulse inhibition. (**A**) Acoustic startle response at baseline (no prepulse) and in response to the prepulses of 69 dB, 73 dB, and 83 dB. Baseline: 0 hits *n* = 12, 1 hit, *n* = 31, 2 hits *n* = 29, 3 hits *n* = 11. 69 dB: 0 hits *n* = 13, 1 hit, *n* = 30, 2 hits *n* = 29, 3 hits *n* = 12. 73 dB: 0 hits *n* = 12, 1 hit, *n* = 31, 2 hits *n* = 32, 3 hits *n* = 13. 83 dB: 0 hits *n* = 13, 1 hit, *n* = 30, 2 hits *n* = 29, 3 hits *n* = 11. (**B**) Percentage of prepulse inhibition in response to the prepulses of 69 dB, 73 dB, and 83 dB. 69 dB: 0 hits *n* = 13, 1 hit, *n* = 27, 2 hits *n* = 29, 3 hits *n* = 11. 73 dB: 0 hits *n* = 13, 1 hit, *n* = 29, 2 hits *n* = 29, 3 hits *n* = 11. 83 dB: 0 hits *n* = 13, 1 hit, *n* = 29, 2 hits *n* = 30, 3 hits *n* = 12. Data is expressed as mean ± SEM and analyzed using Two-Way Mixed ANOVA. **** *p* < 0.0001.

To evaluate deficits in sensory gating, we performed the prepulse inhibition test. Startle response at baseline, and after prepulses of 69 dB, 73 dB, and 83 dB did not differ significantly between hits (F_3,87_ = 0.46, *p* = 0.71, Figure 5A). A main effect of prepulse was also identified for prepulse inhibition, showing that the percentage of prepulse inhibition increased as the prepulse intensity increased (F_1,87_, 226.7 = 18.35, *p* < 0.0001). Hits did not affect the percentage of prepulse inhibition (F_3,243_ = 0.20, *p* = 0.90, Figure 5B).

Analysis of raw data identified a main effect of genotype for the startle response at baseline, where *CNTNAP2*−/− presented with a lower startle than WT mice (F_1,75_ = 5.16, *p* = 0.026). No significant differences were identified for startle response to the prepulse of 69 dB, 73 dB, or 83 dB (*p* > 0.05). Regarding prepulse inhibition, a main effect of genotype demonstrated that *CNTNAP2*−/− mice presented with a lower prepulse inhibition for the pulse of 69 dB when compared to wild-type mice (F_1,73_ = 7.71, *p* = 0.0333). A main effect of treatment showed that Poly IC decreased PPI at 83 dB in all groups (F_1,76_ = 10.74, *p* = 0.0016).

## 3. Discussion

In the present study, we aimed to evaluate whether sex, genetic background, and environment could have a synergic effect, causing a more severe autistic-like phenotype than exposure to any of these factors in isolation. The interaction among these different factors might account for the male bias, where this sex appears to be more sensitive to the influence of genetic [18] and environmental factors [19,20] in the disruption of development. To evaluate the three-hit theory (genetic, environment, and sex), we used, as the environmental factor, the model of maternal immune activation through the administration of Poly IC during gestation, and as a genetic factor, the *CNTNAP2* gene knockout mice. The maternal immune activation model of disruption of neurodevelopment has been extensively studied as an environmental model to induce an autistic-like phenotype in the offspring of rodents and non-human primates [9,21,22,23]. Different dosages and timepoints for treatment with the immune stimulus cause a diverse spectrum of phenotypes in the animals [24,25,26]. Additionally, MIA can have dimorphic effects in the offspring, causing a more severe phenotype in males than in female mice [11]. Another important factor in the pathophysiology of autism is genetic susceptibility. One of the genes linked to ASD is the contactin-associated protein-like 2. Mutations in the *CNTNAP2* gene are linked not just to autism but to other comorbidities such as epilepsy, intellectual disability, and hyperactivity in humans. The mouse model of *CNTNAP2* knockout presents with an autistic-like phenotype characterized by deficits in communication, sociability, stereotyped behavior, cognitive inflexibility, and epilepsy. However, most of the characterization of the *CNTNAP2* knockouts has been made in male mice. A study that also evaluated females identified a more severe phenotype in males, which characterizes a dimorphic presentation of the phenotypes [18]. Here, we performed several behavior tests to evaluate if deficits in communication, stereotyped and repetitive behavior, sociability, working memory, and sensory gating could be affected by a synergic effect of sex, MIA, and *CNTNAP2* deficiency.

### 3.1. Maternal Inflammatory Response

To establish if wild-type and *CNTNAP2*−/− mice presented a similar immune response, we measured the levels of several cytokines and chemokines in the maternal plasma 24 h after Poly IC administration to pregnant dams. At the 24 h timepoint, IP-10 was the only target measured that was significantly increased. Both knockout and wild-type mice presented a similar response to poly IC for all the targets measured. The absence of an effect of Poly IC in most targets measured may be because the peak of the increase in those cytokines and chemokines after Poly IC administration occurs earlier than 24 h [27,28,29]. Additionally, our data presents high variability that, together with the low sample size, may translate into low statistical power.

### 3.2. Behavioral Phenotypes Altered by Gene × Environment × Sex Interactions

Deficits in communication were evaluated in the isolation-induced vocalization test. The number of ultrasonic vocalizations performed by the pups at postnatal day 7 decreased as the number of hits increased, suggesting a cumulative effect of the three hits in the development of this phenotype in the offspring. This data corroborates a previous report where Schaasma et al. demonstrated that MIA at GD 7 with a bacterial agent (lipopolysaccharide—LPS) also decreased the number of vocalizations in a hit-dependent manner, as increasing the number of hits decreased the total number of USVs performed by the animals [30]. These data combined with our current results suggest that both viral and bacterial immune responses during gestation present similar consequences in vocal development in mice, which is dependent on sex and genetic background.

Repetitive and stereotyped behaviors are key features of ASD. To investigate if the severity of this phenotype could be influenced by the hits, we performed grooming and marble-burying tests on the offspring. Hits only affected the performance of the mice in the grooming test, causing a more severe phenotype in the three-hit group when compared to 0 or one hits. In the marble-burying test, however, the number of hits did not affect the animal’s performance in the test. These data indicate that the different areas of the brain responsible for specific stereotypic behaviors may be affected in distinct ways by the factors that the animals were exposed to in the study. Previous reports showed that marble-burying behavior is not altered in male *CNTNAP2* KO when compared to wild-type mice [31,32]. To our knowledge, there are no reports of the performance of female *CNTNAP2* KOs in the marble-burying test. In our study, female knockouts buried almost half as many marbles as female wild-type mice, independent of the treatment, which might explain why we did not see an increase in marbles buried in the one- hit and two-hit groups (Supplementary Table S1).

Sociability deficits are a core feature of ASD. To assess social behavior, we performed the three-chamber test. Social preference was affected by hits, where the three hits presented with increased social preference when compared to the 0 and one-hit groups. Previous studies have consistently shown that *CNTNAP2* KOs present decreased social preference when compared to control mice [15,33,34]. Also, Haddad and collaborators demonstrated that the superimposition of maternal immune activation on *CNTNAP2* heterozygous and knockout rats did not affect social preference or novelty preference in the three-chamber test [35]. In our study, increased sociability in response to hits may have been influenced by repeated testing. Repeated testing can cause decreased exploratory behavior, which could have differently affected mice depending on sex and genotype [36,37].

### 3.3. Behavioral Domains Unaffected by Multi-Hit Exposure

Working memory can be impaired in autistic children and is also impaired in animal models of ASD [38,39]. The percentage of alternations was calculated in the Y-maze test as a reading of working memory deficits. In our study, the percentage of alternations was not altered by hits. Penagarikano et al. showed that male *CNTNAP2* KOs have a decreased percentage of alternations when compared to WT mice [15]. In our study, the percentage of alternations decreased as the hits increased. However, the decrease in alternation did not reach statistical significance. Another parameter that can be evaluated in the Y maze test is the animals’ activity level. Here, we observed no difference between the different hits regarding the total number of entries in the Y-Maze arms.

Deficits in auditory processing are commonly reported in ASD [40]. The preclinical evaluation of sensory gating deficits can be done by an assessment of prepulse inhibition of startle response [41,42]. Our data recapitulates the literature with a decrease in startle response and an increase in prepulse inhibition as the prepulse intensity increases. However, hits had no effect on any of the outputs measured in the test. Previous reports show that sensory gating deficits may be species-specific in *CNTNAP2*−/− rodent models. Adult *CNTNAP2*−/− rats present a higher startle response and decreased prepulse inhibition when compared to the wild type [42,43], while *CNTNAP2*−/− mice do not present alterations in sensory gating in the prepulse inhibition test [15]. Our data suggest that MIA and sex did not interact with the genotype to induce sensory gating deficits, which may have occurred based on the variability in the groups represented in the different hits.

## 4. Materials and Methods

### 4.1. Mice

*CNTNAP2* homozygous knockouts used in this study were originally obtained from The Jackson Laboratory (Stock #017482, Bar Harbor, ME, USA) and used to establish an in-house breeding colony at the University of Texas Health San Antonio. Background strain-matched C57BL/6 control mice were also bred in-house. All animals were group-housed (2–5 mice per cage) in standard cages within an integrated ventilation caging system. Food and water were provided at libitum. Animals were maintained on a 12 h light/dark cycle. Animal care and use were conducted according to the Guide for the Care and Use of Laboratory Animals, Eighth Edition (NRC). Protocols were approved by the Institutional Animal Care and Use Committee at the University of Texas Health San Antonio.

### 4.2. Maternal Immune Activation (MIA)

Polynosinic: polycytidylic acid was used to induce MIA as previously described. Females were checked daily for the presence of seminal plugs after being paired with males from the same genotype. The presence of the plug represented gestational day 0.5 (G0.5). Pregnant dams were randomly assigned to treatment groups. Poly IC or Saline treatment was administered at gestational day 12.5 (GD12.5). Poly IC (Sigma Aldrich, St Louis, MO, USA) was diluted in 0.9% sodium chloride and intraperitoneally injected into pregnant mice at a dose of 20 mg/kg. Control groups were treated with Saline. After treatment, each dam was returned to its cage and left undisturbed until pups were born. All pups were weaned at postnatal day 21 (PD21) and housed with same-sex littermates.

### 4.3. Tissue Collection and Multiplex Assay

A batch of dams was used to establish the different genotypes’ immune responses. 24 h following Saline or Poly IC injections, pregnant dams were humanely euthanized. Blood was collected and centrifuged for 10 min at 1500× *g* at 4 °C for plasma isolation. Plasma was snap frozen in liquid nitrogen and stored at −80 °C until homogenization. Cytokine and chemokine proteins were measured using a Luminex-based multiplex array according to the manufacturer’s instructions (ProcartaPlex Mouse Cytokine/Chemokine, eBioscience; intra-assay CV < 5%). Samples were centrifuged for 10 min at 4 °C at 14,000× *g*. Targets measured were IFN-γ, IL-1β, IL-4, IL-6, IL-10, IP-10, MCP-1, and TNF-α. Results from the dam’s plasma are normalized to % of control (WT Saline) to avoid batch variability.

### 4.4. Ultrasonic Vocalization Test

Isolation-induced ultrasonic vocalizations (USVs) were recorded at postnatal day 7 (PD7) as previously described. Pups were separated from the dam and littermates and placed into a clean container made of glass (200 mL beaker) containing fresh bedding material at room temperature. After 10 min of isolation, an Ultramic200K microphone (Dodotronic Ultramic 200 K, Dodotronic, Castel Gandolfo, RM, Italy) was used to record the vocalizations in a sound-attenuating chamber for 5 min. Vocalizations were recorded using SeaWave—Sound Emission Analyzer Wave Edition Software 2 (CIBRA, Pavia, Italy) at a sampling rate of 200 kHz. After the recording was done, the pup was returned to its home cage. Analysis of recorded USVs was done by using UltraVox XT 3.2 (Noldus Information Technology, Leesburg, VA, USA) to count the number of vocalizations produced by each pup.

### 4.5. Grooming

Self-grooming behavior was evaluated according to Malkova et al. (2012) [7]. Five-week-old mice were habituated in a plastic beaker (16 cm diameter × 25 cm height) for 10 min. Following habituation, a grooming test was performed for 5 min. Grooming behavior was scored as the time that the mice spent licking, touching, scratching, or biting paws, face, body, or tail.

### 4.6. Marble Burying

Marble-burying behavior was assessed in six-week-old mice as previously established. Animals were placed in the testing cage for habituation for 10 min (cage size: 8 × 18.5 × 10, bedding depth: 4 cm). After habituation, the mouse was briefly returned to the home cage while 20 marbles were placed in the test cage in a 4 × 5 grid on top of the bedding. The mouse was returned to the testing cage, and at the end of the 10 min of exploration, the number of marbles buried was counted. The criterion for marbles to be considered buried was that each marble had to be at least 2/3 covered by bedding.

### 4.7. Y Maze

Eight-week-old mice were placed in the Y maze and allowed to explore for 8 min to assess alternation behavior as previously described. The performance of each animal was recorded from an overhead camera. The total number of entries and the sequence of arm entries were scored. An entry was scored only when the full body of the mouse was at least 2 cm deep into the maze arm. Spontaneous alternations were considered when the mouse entered each of the three arms sequentially without repeating arms already visited. The percentage of alternations was calculated as the total number of alternations/total arm entries −2 × 100.

### 4.8. Three-Chamber Test

Eight-week-old mice’s sociability was assessed in the three-chamber test. An acrylic box (63 × 41 × 26 cm) divided into three equal chambers was used to perform the test. In the 10 min of habituation, mice were able to freely move between chambers through a 5 × 5 cm opening between all chambers. After habituation, the mice were returned to their home cage while the box was cleaned. An unfamiliar (novel conspecific) mouse of the same sex was placed under a wire cup inside one of the outer chambers, while in the opposite outer chamber, there was an empty wire cage. Mice were placed in the center chamber and allowed to freely explore for 10 min. The movement of the test mouse was video recorded by an overhead camera. In the second part of the test, a new novel conspecific mouse was placed under the wire cup in one outer chamber, while the mouse that served as the first novel conspecific remained in the other chamber. The locations of the familiar and novel stranger mice were randomly selected. For 10 min, the mice were able to interact with familiar and novel mice, and the time of interaction was recorded. The social preference index was calculated as [timesocial × 100/(timesocial + timenon-social)], while the novelty preference index was calculated as [timenovel × 100/(timefamiliar + timenovel)] and represented as %.

### 4.9. Prepulse Inhibition

The prepulse inhibition (PPI) test was conducted in an acoustic chamber (SR-LAB, San Diego Instruments, San Diego, CA, USA), as previously described by Donegan et al. (2018) [44]. Briefly, mice were placed in a sound-attenuated chamber and allowed to acclimatize to the background noise (65 dB) for 5 min. The startle response was induced by a pulse of 120 dB for 40 ms repeated ten times. Prepulse inhibition was induced using prepulses of 69 dB, 73 dB, and 81 dB that lasted 20 ms. The startle response was analyzed using SR-LAB Analysis Software (San Diego Instruments, San Diego, CA, USA) and measured from 10 to 80 ms after the onset of the startle pulse. PPI was calculated as the percentage of decrease in an increase/decrease in startle response in response to the prepulse following the formula: %PPI = [(startle response − prepulse startle response)/startle response] × 100.

### 4.10. Data Analysis

To investigate the three-hits hypothesis (genotype × environment × sex), the data were organized into four categories (zero to three hits) as previously described. Briefly, the data was organized as follows: 0 hits (WT females Saline), 1 hit (WT female Poly IC, WT male Saline, and *CNTNAP2* KO female Saline), 2 hits (WT male Poly IC, *CNTNAP2* KO female Poly IC, and *CNTNAP2* KO male Saline), and 3 hits (*CNTNAP2* KO males Poly IC). The hits data were analyzed with a one-way analysis of variance (ANOVA). Prepulse inhibition test data were analyzed by a two-way Mixed ANOVA with Geisser–Greenhouse’s correction to assess the effect of increasing the prepulse. To better understand how the hits affected the output of the behavior paradigms, we also analyzed the raw data with a three-way ANOVA with between-subject factors of genotype, treatment, and sex. This analysis can be found in Supplementary Table S1. The groups were WT male Saline, WT male Poly IC, *CNTNAP2* KO male Saline, *CNTNAP2* KO male Poly IC, WT female Saline, WT female Poly IC, *CNTNAP2* KO female Saline, and *CNTNAP2* KO female Poly IC. Cytokine data was analyzed by a two-way ANOVA. For the cytokines analysis, the groups made of pregnant dams were WT Saline, WT Poly IC, *CNTNAP* KO Saline, and CNTNAP2 KO Poly IC. Data are represented as mean ± standard error mean (SEM). Tukey’s post hoc test was used to analyze interactions between genetic background, treatment, and sex when appropriate. Chauvenet’s test was used to identify spurious data in each group before the hits analysis when the sample size was ≥10. Graphpad Prism 9.5.1 (La Jolla, CA, USA) was used for data analysis. *p*-value < 0.05 was considered statistically significant.

### 4.11. Use of Generative AI

The authors used ChatGPT 5.2 to assist in the generation of the graphical abstract based on content and prompts reflecting the authors’ original conceptual ideas. The final decision-making and responsibility for the content remains with the authors.

## 5. Conclusions

The current study examined the interplay among sex, genetic background, and environmental factors in the manifestation of an autistic-like phenotype in mice. By utilizing the three-hit model incorporating maternal immune activation (MIA), *CNTNAP2* deficiency, and sex, we aimed to elucidate whether the convergence of these factors exacerbates the severity of ASD-related behaviors. Our findings highlight the nuanced nature of this interaction, indicating behavior-specific effects. Deficits in communication, repetitive behaviors, and sociability were significantly influenced by the cumulative hits. Alternatively, deficits in working memory and sensory gating were not affected by hits. Overall, our study contributes to the growing literature on the complex etiology of ASD and underscores the importance of considering multiple factors in preclinical research aimed at unraveling its underlying mechanisms.

## Figures and Tables

**Table 1:** Levels of cytokines and chemokines in maternal blood. Plasma concentration of cytokines and chemokines was assessed 24 h after Poly IC administration into dams on gestational day 12.5. *n* = 3–4 dams.

Target	% of Control (WT SAL)				Interaction	Main Effects	
	WT Saline	WT Poly IC	*CNTNAP2* KO−/− Saline	*CNTNAP2* KO−/− Poly IC		Genotype	Treatment
IFNγ	100 ± 0	626.19 ± 1018.8	100 ± 0	122.25 ± 38.55	n.s.	n.s.	n.s.
IL-1β	100 ± 51.61	1045.75 ± 1685.65	169.96 ± 111.48	525.13 ± 655.54	n.s.	n.s.	n.s.
IL-4	100 ± 6.60	112.10 ± 31.18	98.82 ± 6.40	128.79 ± 57	n.s.	n.s.	n.s.
IL-6	100 ± 45.74	58.13 ± 32.92	80.77 ± 63.69	119.07 ± 53.73	n.s.	n.s.	n.s.
IL-10	100 ± 53.43	280.93 ± 435.35	75.38 ± 27.98	147.87 ± 96.79	n.s.	n.s.	n.s.
IP-10	100 ± 68.33	250.76 ± 159.70	68.07 ± 40.38	237.11 ± 136.61	n.s.	n.s.	*p* = 0.03 *
MCP-1	100 ± 68.18	129.68 ± 124.65	99.19 ± 87.67	96.87 ± 21.41	n.s.	n.s.	n.s.
TNF-α	100 ± 45.07	438.78 ± 586.06	97.02 ± 84.01	68.86 ± 19.20	n.s.	n.s.	n.s.

Data is expressed as % of the WT SAL group and analyzed using a two-way ANOVA test followed by Tukey’s post hoc test. * *p* < 0.05. n.s. = no significant difference. WT = wild type. *CNTNAP2* = contactin-associated protein-like 2.

## Data Availability

The original contributions presented in this study are included in the article/Supplementary Material. Further inquiries can be directed to the corresponding author.

## Supplementary Materials

The following supporting information can be downloaded at: https://www.mdpi.com/article/10.3390/ijms27062566/s1.
