# Supplementary material for: Analysis of Gene, Environment, and Sex Interaction in the Development of Autistic-like Phenotype in Mice
Source: Int J Mol Sci. 2026 Mar 11;27(6):2566. doi: 10.3390/ijms27062566 (PMC13026612; doi:10.3390/ijms27062566)
Supplement: Supplementary file 1 [file ijms-27-02566-s001.zip › ijms-4151721-supplementary.pdf]

Behavior	Male				female				Interaction	main effects		
	WT Sal	WT Poly IC	CNTNAP -/- Sal	CNTNAP2 -/- Poly IC	WT Sal	WT Poly IC	CNTNAP -/- Sal	CNTNAP2 -/- Poly IC		genotype	treatment	sex
USV (n)	178.5 ± 138	105.83 ± 81.42	156.75 ± 129.73	45.29 ± 46.67	216.5 ± 125.55	82.33 ± 90.02	93 ± 135.77	94.33 ± 59.94	n.s.	n.s	p=0.02	n.s.
Grooming (sec)	30.36 ± 19.46	20.18 ± 20.02	72.7 ± 23.73	73.27 ± 26.87	26.43 ± 25.84	36.78 ± 34.24	55.54 ± 24.35	56.45 ± 33.16	n.s.	p<0.001	n.s.	n.s.
Marble buried (n)	3.55 ± 2.58	4.36 ± 3.96	0.9 ± 0.1.73	4.59 ± 3.28 #	4.07 ± 3.02	5.67 ± 3.35	2.07 ± 2.13	1.82 ± 1.94	gen x treat x sex (p<0.05)	p < 0.001	p<0.05	n.s.
Total Arm entry (n)	34.64 ± 9.82	36.67 ± 5.94	50.4 ± 13.41*	51.93 ± 8.16*** +	43.07 ± 9.37	43.44 ± 10.97	51 ± 8.37 **	49.08 ± 11.86*	gen x sex (p=0.04)	p< 0.0001	n.s.	n.s.
Alternation (%)	53.52 ± 10.27	54.85 ± 7.82	51.91 ± 10.59	47.78 ± 7.10	54.43 ± 6.27	52.61 ± 5.40	50.45 ± 7.61	42.92 ± 6.96	gen x treatment (p =0.0011)	n.s.	n.s.	n.s.
Social Preference (%)	55.16 ± 6.45	62.66 ± 3.94	65.37 ± 6.28	63.57 ± 11.21	53.48 ± 9.19	61.32 ± 8.81	56.84 ± 7.54	60.03 ± 9.35	n.s.	n.s.	p=0.0358	p=0.0203
Social Novelty Preference (%)	66.48 ± 10.53	57.18 ± 12.28	55.55 ± 9.12	54.47 ± 11.04	51.24 ± 12.30*	59.32 ± 5.05	50.82 ± 11.26*	61.16 ± 14.90	sex x treatment (p=0.0053)	n.s.	n.s.	n.s.
Startle response (baseline)	786.99 ± 459.09	841.08 ± 436.98	640.77 ±203.22	452.61 ± 231.79	663.54 ± 248.21	519.1 ± 117.17	602.02 ± 292.96	497.37 ± 236.46	n.s.	p= 0.0260	n.s.	n.s.
Startle response(69dB)	622.12 ± 403.49	503.79 ± 228.76	491.76 ± 209.25	571.57 ± 453.15	554.34 ± 167.90	461.53 ± 212.25	450.51 ± 222.25	425.13 ± 149.26	n.s.	n.s.	n.s.	n.s.
Startle response(73dB)	355.20 ± 178.59	259.72 ± 141.21	297.50 ± 152.80	288.87 ± 250.36	333.21 ± 127.86	208.54 ± 100.54	388.76 ± 179.07	323.12 ± 304.48	n.s.	n.s.	n.s.	n.s.
Startle response(83dB)	164.24 ± 105.68	193.78 ± 162.46	164.25 ± 107.50	170.74 ± 133.68	150.85 ± 68.29	150.4 ± 89.67	203.35 ± 100.92	177.92 ± 155.11	n.s.	n.s	n.s.	n.s.
Prepulse inhibition 69dB (%)	15.41 ± 14.96	36.66 ± 20.41	1.21 ± 25.06	2.91 ± 19.01	16.54 ± 32.73	11.54 ± 36.94	14.83 ± 35.82	2.17 ± 45.53	n.s.	p = 0.0333	n.s.	n.s.
Prepulse inhibition 73dB (%)	44.93 ± 15.51	48.13 ± 12.92	36.52 ± 29.09	48.71 ± 20.72	38.29 ± 20.38	51.68 ± 17.63	25.50 ± 30.42	25.96 ± 57.81	n.s.	n.s.	n.s.	n.s.
Prepulse inhibition 83dB (%)	59.86 ± 12.44	35.31 ± 22.04	48.23 ± 16.20	38.66 ± 21.43	49.36 ± 19.11	20.31 ± 49.77	44.63 ± 24.34	38.28 ± 22.95	n.s.	n.s.	p= 0.0016	n.s.

Supplemental Table S1. Raw data analysis for all behavior tests. Data from the ultrasonic vocalization (USV), marble burying, Y-MAZE (Total arm entry and alternations), and Three Chamber test (Social preference and Social Novelty Preference) are represented as mean ± SEM and analyzed with a Three Way ANOVA followed by a Tukey postdoc. Data from the prepulse inhibition test (Startle response at baseline, 69dB, 73dB, 83 dB, and prepulse inhibition at the 69dB, 73dB, and 83dB prepulse) are represented as mean ± SEM and analyzed with a Three Way ANOVA. * vs Male WT Saline, + vs Male WT Poly IC, # vs Male CNTNAP2 -/- Saline. * $p < 0.05$, ** $p < 0.01$, *** $p < 0.001$.
